# The Clinical Diagnosis of Nasal Discharges

**Published:** 1899-07

**Authors:** W. E. A. Wyman


(To be continued.)

L. A. MERILLAT, V.S.

THE CLINICAL DIAGNOSIS OF NASAL DISCHARGES. On the whole, nasal discharges as a determining factor in regard to the nature, extent, and location of the diseased area from which they exude are unreliable.

It is to be admitted that a unilateral, greenish-yellowish, glutinous exudate forming into crusts about the margin of the nostril is suggestive of glanders, but the mere fact of finding such a unilateral discharge does not entitle to the diagnosis of glanders, as discharges of a similar nature are exhibited also in other diseases of the nasal passages. Therefore, with that point as a basis—that is, the wanting reliability of nasal discharges as a decisive diagnostic feature—certain instruments have been constructed to aid in locating the site of the diseased area. These instruments, while very scientific, very ingenious, and very costly, are mainly of value for demonstrations at the clinics of such schools as we may hope to have in our country some day. At the same time, the average practitioner will have but little use for them. The ordinary nasal speculum, or, just as well, the thumb and index finger dexterously employed, are quite satisfactory. As a matter of interest may be mentioned the Priestley-Smith lamp or Bayer's electric reflector, the panelectoscope of Leiter, together with the nasal tubes of Polansky and Schindelka.

Below will be discussed not only the various exudates arising within the nasal passages, but also such points which are of importance to the surgeon as a matter of differentiation. The writer takes this opportunity to remind the ultra-scientific reader of the JOURNAL that the contents of the pages devoted to the Department of Surgery are primarily intended for those who, like most of us, graduated at a time and under conditions quite different from what we find to-day. This statement, it is hoped, will be accepted not as one which throws a slur upon the graduates of some years of standing, but one which intimates the necessity—the absolute necessity—of post-graduate work and more extensive under-graduate work than has been meted out in the past.

Nasal discharges may originate in the lungs, bronchi, trachea, larynx, pharynx, guttural pouches, sinuses of the head, nasal cavity, and, finally, in the annexes of the buccal cavity. Before going into the details of the various diseases and their exudates, nasal exhalations will be briefly dealt with. The temperature, the odor, and the force of the air-current as it leaves the nostril enables us frequently to make important clinical deductions. It is supposed that previous practice, such as should be acquired in the clinics of our schools, or as most of us have acquired at the expense of our customers, has rendered the hand of the one conducting the examination sufficiently expert to detect at once whether the temperature is a normal one. In diseases with lethal exit the temperature of the expired air is below normal, also in internal hemorrhages, collapse, anæmia, etc.—an important prognostic point. In estimating the force of the air as it leaves the nostril, it is best done by closing first one and then the other nostril; one is able to recognize stenosis of the nasal passage, the subsequent examination to reveal whether the offending agent is a neoplasm, swollen mucous membrane, fractured wall of the nasal canal, or due to disease of the turbinated bones, etc.

The odor can almost invariably be traced to some necrotic animal tissue along the respiratory or digestive tract. While the olfactory nerves of some especially fortunate practitioners are so wonderfully trained as to detect and locate the trouble at once, the majority of us, nevertheless, would have to continue the examination before reaching a final verdict. Decaying teeth, no doubt, are most frequently the cause of a fetid odor, which at the same time is characteristic; the odor accompanying chronic catarrh of the nose, chronic catarrh of the guttural pouches, empyema of the frontal and maxillary sinuses, alveolar periostitis, croupous exudates in the nasal canal, larynx, pharynx, trachea, and bronchi, also disintegrating tumors, as a sarcoma, angioma, or carcinoma, give rise to odors the intensity and quality of which depend on the amount of necrotic tissue present; finally, the odor met with in gangrene of the lungs is peculiar and diagnostic. All important is, of course, the source of the odor. In order to get at it, it is to be decided whether it is uni- or bilateral. By placing the hand between the nostrils, in line with the septum nasi, the odor can readily be traced to one or the other nasal canal, or is found to emanate from both nostrils. Should the odor be equally strong from both orifices, it is reasonable to presume that its origin is somewhere in the pharynx, larynx, trachea, bronchi, or lungs, further examination being then required to locate the exact source. Unilateral odor originates in the sinuses, guttural pouch, or nasal cavity of that side. The examination of the buccal cavity and its saliva might place the source of the odor there. Diseases of the teeth, alveolar periostitis, decomposing food, ulcerations, etc., would serve as an explanation. As previously stated, the odor of the saliva may contribute materially in locating its origin. It is to be remembered that the saliva should not be tested immediately following a cough. Granting, then, that the odor of the saliva is tested at the proper time, we find it inodorous (as a rule) in such diseases of the trachea, bronchi, lungs, sinuses of the head, guttural pouches, which ordinarily produce fetid odors. Decomposing food, carious teeth, alveolar periostitis, and fistulæ into the mouth give the saliva a more or less fetid odor.

W. E. A. Wyman, M.D.V., V.S.

(To be continued.)

### Surgical Items.

Iodoform must be regarded as one of the most valuable antiseptics for veterinary surgery. It promotes granulations, limits the exudates, relieves pain, is practically non-toxic, and besides, unlike other dry antiseptics, it does not prevent immediate union when caught between the edges of wounds.—L. A. M.

No veterinary hospital should be without a formaldehyde sterilizer. Instruments, bandages, wadding, etc., can be sterilized in from ten to fifteen minutes, and with more convenience than with dry heat or boiling. The effectiveness of formaldehyde gas in this connection is no longer an experiment.—*Id.*

When a molar of a horse is extracted the crown of its antagonist should be removed to prevent packing of fibrous food into the cavity. Very young horses should be made an exception to this rule.—*Id.*

The chief objection to "standing operations" is found in the principle that a surgeon should have his patient and seat of operation under his full and perfect control at all times.—McKillip.

In all septic processes it is well to remember the beneficial effects of iron preparations. Even in the minor cases, such as whitlow, etc., it is of great value.—*Int. Journ. of Surgery.*

When in doubt in regard to the diagnosis between abscess and aneurism, compress the artery above. If an abscess, the pulsation will reappear at once; if an aneurism of fair size, the pulsation will only recur after the sac has been filled again.—*Id.*

